# Overtures to Peace

**Published:** 1885-12

**Authors:** 


OVERTURES TO PEACE.—We learn unofficially, but from an entirely reliable source, that at a meeting of the International Congress Executive Committee, held in New York recently, it was determined to invite "the old committee" to join in the work of organizing the Congress ; and that the vacancies occasioned by recent resignations were not filled—purposely—in order that the old committee might have a voice in the matter, and aid in the work if they will.

Without discussing the wisdom, propriety, expediency, necessity, or taste of this action, we give our readers the information, with the single remark that a refusal on the part of the old committee to co-operate in the work of organization, now that the invitation is extended to them formally—we may say, for *the second time*, they being all the while *part of* the committee—will throw the responsibility of a failure on them, should there be a failure, and the world should know it. Our informant says "the affairs of the Congress are brightening daily."

We sincerely trust that all dissensions will now cease, and that the whole medical profession of America will unite in the endeavor to make the Congress a success.

The 7th of September, 1887, has been set for the opening of the Congress.

